# H5N1 Influenza Virus, Domestic Birds, Western Siberia, Russia

**DOI:** 10.3201/eid1207.051338

**Published:** 2006-07

**Authors:** Alexander M. Shestopalov, Alexander G. Durimanov, Vasily A. Evseenko, Vladimir A. Ternovoi, Yury. N. Rassadkin, Yulya V. Razumova, Anna V. Zaykovskaya, Sergey I. Zolotykh, Sergey V. Netesov

**Affiliations:** * State Research Center Virology and Biotechnology Vector, Koltsovo, Novosibirsk, Russia

**Keywords:** Avian influenza, H5N1, influenza, domestic birds, Siberia, letter

**To the Editor:** Highly pathogenic H5N1 avian influenza virus caused disease outbreaks in poultry and wild birds in several Asian, European, and African countries from 2003 to 2006. This virus caused >90 human deaths in Vietnam, Thailand, People's Republic of China, Indonesia, Turkey, Iraq, and Cambodia (*1**–**3*). Hemagglutinin (HA) and neuraminidase (NA) genes of this virus were derived from the Gs/Gd/1/96-like lineage, and 6 genes that encode internal viral proteins were derived from other lineages (*1*).

Highly pathogenic H5N1 virus genetically related to the A/Chicken/Shantou/4231/03 (People's Republic of China) isolate caused disease outbreaks in poultry in Japan from the end of December 2003 to March 2004 (*4*). In May and June 2005, highly pathogenic H5N1 virus was isolated from migratory birds during disease outbreaks near Lake Qinghai in western People's Republic of China. HA, NA, and nucleoprotein genes of the Qinghai virus were closely related to H5N1 virus A/Chicken/Shantou/4231/03 isolated in People's Republic of China in 2003. Five other viral genes (matrix, PA, PB1, PB2, and nonstructural protein) were closely related to an H5N1 Hong Kong Special Administrative Region, People's Republic of China 2004 isolate (A/Peregrin falcon/HK/D0028/04) and H5N1 virus A/Chicken/Shantou/810/05 isolated in People's Republic of China in 2005 (*5**,**6*).

In July 2005, domestic poultry began to die in the village of Suzdalka in western Siberia, Russia (Dovolnoe County, Novosibirsk region). Autopsies showed serious alterations in all internal organs tested. Approximately 95%–100% of the lungs were affected, and all serous membranes showed petechial and confluent hemorrhages. The highest concentration of hemorrhages was in the pericardium.

Organs from 3 birds (1 turkey and 2 chickens) that had died during this outbreak were further analyzed. Homogenates of lungs, kidneys, and spleens were tested by hemagglutination inhibition (HI) assay. The highest titers, 32 and 16, were observed in the spleen of the turkey and kidneys of the chickens, respectively. H5 influenza A virus was identified in a homogenate of turkey spleen by conventional HI assay (*7*) with a panel of reference antisera.

For the identification of NA subtype, RNA was isolated from turkey spleen homogenate and synthesis of viral cDNA was performed as previously described (*7*). Amplification by polymerase chain reaction (PCR) and sequencing of an NA gene fragment were performed with in-house primers (sequences of primers are available on request). The nucleotide sequence obtained (547 bp, GenBank accession no. DQ231243) showed 100% identity with the NA gene of H5N1 viruses isolated in People's Republic of China in 2005 (e.g., A/Great black-headed gull/Qinghai/1/05) (*5**,**6*).

Homogenates of bird organs (turkey spleen and chicken kidneys) were injected into the allantoic cavity of 10-day-old embryonated chicken eggs. Three hemagglutinating agents were isolated (titers 1,024–2,048) and identified as H5 influenza A virus (A/Turkey/Suzdalka/Nov-1/05, A/Chicken/Suzdalka/Nov-11/05, and A/Chicken/Suzdalka/Nov-12/05) by reverse transcription–PCR and sequencing (isolation of RNA from allantoic fluid and synthesis of virus cDNA were performed as previously described [*7*]). PCR amplification and sequencing of a fragment of the HA gene were performed with an in-house primer set for the H5 gene (available on request). Phylogenetic analysis of nucleotide sequences obtained (GenBank accession nos. DQ231242, DQ231241, and DQ231240) indicated that western Siberian 2005 isolates belong to the Gs/Gd/1/96-like lineage and form a cluster with H5N1 viruses isolated from migratory birds in the People's Republic of China in 2005 (*5*), from poultry in Japan in 2004 (*4*), and from poultry and humans in Asian countries in 2003 and 2004 (*1*) (Figure). Deduced amino acid HA cleavage site sequences of all isolates (PQGERRRKKR/GL) corresponded to highly pathogenic Asian H5N1 influenza virus variants (*5**,**6*).

**Figure:**
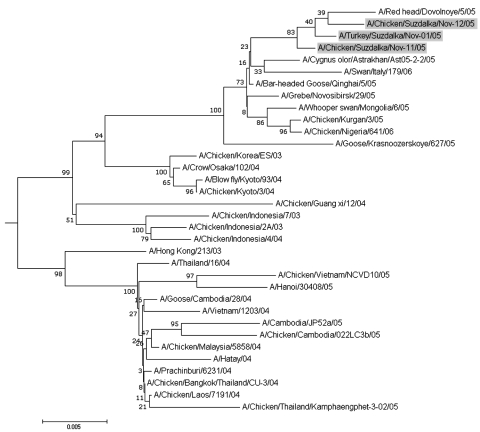
Phylogenetic tree of H5 hemagglutinin genes of influenza A viruses. The 3 H5 western Siberian 2005 viruses isolated in this study are shaded. Phylogenetic analysis was performed by the neighbor-joining method with the Molecular Evolutionary Genetic Analysis 2 program (Center for Evolutionary Functional Genomics, Tempe, AZ, USA). Scale bar indicates relative value of distance in matrix normalized units. Numbers indicate tree divarication.

To test virulence, 10 six-week-old chickens were intravenously infected with isolate A/Turkey/Suzdalka/Nov-1/05 as previously described (*7*). All viruses isolated were highly pathogenic (all chickens died within a day of infection).

We isolated H5N1 influenza virus from the spleen of a turkey that died during an outbreak in poultry in western Siberia in July 2005. HA and NA genes of this virus were closely related to those of H5N1 avian influenza viruses that caused outbreaks in birds in Asian countries from 2003 to 2005 and in Japan in 2003 and 2004. The corresponding isolate, A/Turkey/Suzdalka/Nov-1/05, from turkey spleen was highly pathogenic for chickens in the laboratory intravenous pathogenicity index test. The origin of this H5N1 virus in western Siberia is not known. Migratory birds could have introduced this virus because western Siberia is located on a flyway of wild birds that migrate in the spring from southeastern Asia. Highly pathogenic Asian H5N1 influenza virus in western Siberia demonstrates spread of these Asian viruses into new areas and suggests a larger geographic distribution.

## References

[1] Li KS, Guan Y, Wang J, Smith GJ, Xu KM, Duan L, Genesis of a highly pathogenic and potentially pandemic H5N1 influenza virus in eastern Asia. Nature. 2004;430:209–13. 10.1038/nature02746 15241415

[2] World Health Organization Global Influenza Program Surveillance Network. Evolution of H5N1 avian influenza viruses in Asia. Emerg Infect Dis. 2005;11:1515–21. 16318689 10.3201/eid1110.050644 PMC3366754

[3] Cumulative number of confirmed human cases of avian influenza A/(H5N1) reported to WHO. 2002 [cited 2006 Apr 12]. Available from http://www.who.int/csr/disease/avian_influenza/country/cases_table_2006_02_13/

[4] Mase M, Tsukamoto K, Imada T, Imai K, Tanimura N, Nakamura K, Characterization of H5N1 influenza A viruses isolated during the 2003–2004 influenza outbreaks in Japan. Virology. 2005;332:167–76. 10.1016/j.virol.2004.11.016 15661149

[5] Liu J, Xiao H, Lei F, Zhu Q, Qin K, Zhang X, Highly pathogenic H5N1 influenza virus infection in migratory birds. Science. 2005;309:1206. 10.1126/science.1115273 16000410

[6] Chen H, Smith GJ, Zhang SY, Qin K, Wang J, Li KS, Avian flu: H5N1 virus outbreak in migratory waterfowl. Nature. 2005;436:191–2. 10.1038/nature03974 16007072

[7] World Health Organization. Manual on animal influenza surveillance. Geneva: The Organization; 2002.

